# ‘Mechanistic insights into 5-lipoxygenase inhibition by active principles derived from essential oils of *Curcuma* species: Molecular docking, ADMET analysis and molecular dynamic simulation study

**DOI:** 10.1371/journal.pone.0271956

**Published:** 2022-07-22

**Authors:** Ayushman Gadnayak, Budheswar Dehury, Ananya Nayak, Sudipta Jena, Ambika Sahoo, Pratap Chandra Panda, Asit Ray, Sanghamitra Nayak

**Affiliations:** 1 Centre for Biotechnology, Siksha ‘O’ Anusandhan (Deemed to be University), Bhubaneswar, Odisha, India; 2 ICMR-Regional Medical Research Center, Nalco Square, Chandrasekharpur, Bhubaneswar, Odisha, India; Aligarh Muslim University, INDIA

## Abstract

Inflammation is caused by a cascade of events, one of which is the metabolism of arachidonic acid, that begins with oxidation by the enzyme 5-lipoxygenase. 5-Lipoxygenase (5-LOX) plays an important role in the inflammation process by synthesizing leukotrienes and several lipid mediators and has emerged as a possible therapeutic target for treatment of inflammatory diseases such as asthma and rheumatoid arthritis. Most of the existing 5-LOX inhibitors are synthetic and exhibit adverse side effects. In view of this, there is need to search for an alternate source of 5-LOX inhibitor with minimal side effects. The essential oil of several species of *Curcuma* has received considerable attention in recent times in traditional system of medicine especially for treating various inflammatory disorders. Therefore, the present study was carried out to screen the most potential 5-LOX inhibitors from essential oil components of *Curcuma* species and elucidate their mechanisms of action through computational biology approaches. Twenty-three phytoconstituents derived from the essential oil of *Curcuma* species were docked and their predictive binding energies were calculated to select the best possible ligand for 5-LOX. The top 8 ranked compounds from docking was tested for drug-likeness properties, bioactivity score, and toxicity analysis. The phytoconstituents such as *α*-turmerone, *β*-turmerone, *α*-terpineol and dihydrocarveolshowed the best binding affinity with 5-LOX and displayed favorable physicochemical properties. Molecular dynamics simulation in POPC lipid bilayers was carried out to understand the intrinsic dynamics and flexibility of the 5-LOX (apo) and 5-LOX-complex (*α*-terpineol, *α*-turmerone, *β*-turmerone and dihydrocarveol) systems. The molecular dynamic results showed that these 4 phytoconstituents interacted stably with the 5-LOX active site residues and the important bonds that were observed in the initial ligand docked compounds did not alter during the course of simulation. In general, our integrative computational approach demonstrated that the natural compounds like *α*-turmerone, *β*-turmerone, *α*-terpineol, and dihydrocarveol could be considered for designing specific anti-inflammatory drugs using structure-based drug design.

## 1. Introduction

Inflammation is the body’s natural defensive response to a variety of internal and external stimuli [1]. Long-term chronic inflammation is linked to the development and progression of autoimmune diseases, cancer, cardiovascular and neurological disorders. Overproduction of mediators of the arachidonic acid (AA) cascade, particularly those of the cyclooxygenase (COX) and lipoxygenase (LOX) pathways, causes major inflammatory diseases in humans [2].

The enzyme 5-lipoxygenase (5-LOX) catalyzes the oxidation of arachidonic acid, which in turn reacts with eicosapentanoic acid, resulting in the formation of leukotrienes, the chemical mediators of inflammation [3]. In the biosynthesis of leukotrienes, 5-LOX plays a critical role, and it is perceived that drugs that inhibit 5-LOX will inhibit the generation of inflammatory mediators from the arachidonic acid pathway. As a result, inhibition of 5-LOX enzymes has been established as a rational approach for developing novel anti-inflammatory drugs with a better safety profile.

Synthetic 5-LOX inhibitors such as zileuton exhibits hepatotoxic effects as observed in most other synthetic drugs [4]. Because of this potential constraint, inhibitors alternative to 5-LOX with little or no adverse effects need to be developed. Phytochemicals isolated from plants have shown to suppress the production of the enzyme 5-LOX by interfering with mitogen activated protein kinase (MAPK) and nuclear factor kappa B (NF-κB) signal transduction pathways [5]. Plants used in traditional and ethnomedicine are known for their proven efficacy and minimal adverse effects. In recent times, various research has been carried out utilizing plant-derived essential oils for treating inflammatory disorders. While exploring a plethora of essential oil from different genera, we selected essential oils of *Curcuma* species as their bioactive constituents targeting 5-LOX enzyme have not been extensively studied so far.

The genus *Curcuma* of the family Zingiberaceae includes a group of rhizomatous herbaceous perennial plants native to tropical and subtropical parts of Asia, Australia, and South America [6]. The major bioactive components of the rhizome are nonvolatile curcuminoids and the essential oil [7]. The essential oil of *Curcuma* species possesses anti-inflammatory, anti-cancerous, antioxidant, antimicrobial, antiviral, anti-diabetic, anti-hepatotoxic, anti-diarrheal, carminative, diuretic, anti-rheumatic, antithrombotic, anti-venomous, anti-tyrosinase, hypocholesterolemic, insecticidal and larvicidal activities and many other pharmacological applications [7]. The terpenes and sesquiterpenes isolated from essential oil are good inhibitors of 5-LOX enzyme *in vitro* [8]. The essential oil of *Curcuma* species contains several biologically active compounds, including *ar*-turmerone, *α*-turmerone, *β*-turmerone,*α*-terpineol and dihydrocarveol, which have considerable anti-inflammatory activity and can prove useful for development of new anti-inflammatory drugs [9, 10]. These compounds have been isolated from different *Curcuma* species, including *C*. *brog*, *C*. *aromatica*, *C*. *caesia*, *C*. *malabarica*, and *C*. *zedoaria* [11].

Considering the enormity of research reports on the inflammatory effects of essential oil of *Curcuma* species and the importance of 5-LOX as a therapeutic target in inflammation, we aimed to carry out an *in-silico* evaluation of phytocompounds derived from essential oils of *Curcuma* species against 5-LOX and determine their structure-function mechanism through virtual screening, molecular docking, and molecular dynamics simulation. The results of our computational study would provide a benchmark for specific plant-based inhibitors derived from essential oils against 5-LOX, which may open-up new avenues for the development of novel and more effective anti-inflammatory drugs.

## 2. Materials and methods

### 2.1 Target receptor protein 5-LOX and ligands

A number of three-dimensional structures of un-complexed 5-LOX (UniProtKB: P09917) from *Homo sapiens* (PDB ID: 3O8Y, 3V92, 3V98, 3V99, 6N2W, and 6NCF) have been solved through X-ray crystallography, but all of them harbor a series of mutations. Therefore, in the present study, we downloaded an α-fold derived full-length model of 5-LOX with an overall root mean square deviation (RMSD) of 0.205 Å with that of crystal structure 3O8Y for *in silico* studies. A total of 23 phytocompounds from *Curcuma* species were used collectively as ligands in this study. The three-dimensional structures of ligands were downloaded from the PubChem database and were later optimized using BIOVIA Discovery Studio Visualizer version 4.5 (BIOVIA DSV).

### 2.2 Identification of binding pocket

Prank Web (https://prankweb.cz/), a machine learning based method was used for the prediction of plausible binding pockets of 5-LOX protein [12]. Binding site denotes the distribution of nearby amino acid residues in active pocket and act as catalytic residues [13, 14].

### 2.3 Molecular docking

Glide program was employed for docking calculations of the ligands with the target receptor (5-LOX). Prepared ligands were docked and scored first with the standard-precision (SP) mode of Glide based on OPLS-3e force field. Subsequently, from the top-scored SP poses, each ligand was re-docked with higher-level extra-precision (XP) to eliminate false positives and for good enrichment.

### 2.4 Computing the ligands binding affinity using MM/GBSA

The binding free energy was computed via Schrodinger Prime Molecular Mechanics Generalized Born and Surface Area (MMGBSA) module. Briefly, the binding free energy (ΔG_bind_) was calculated by the addition of the solvation free energy and gas-phase interaction energy, while ignoring the entropy term. The ligand binding affinities can be represented by MM/PBSA. The method involves six energy terms that can be tested individually and improved, the electrostatic term is based on the charges of the receptor as well as the ligand. In these regard in order to improve the polarizable potentials, quantum mechanics (QM) calculations as well as multipole expansions attempts can be taken with improvement in the ΔG bind and depends on the dielectric constant that is needed for the protein. The results of the interaction can be improved by using ϵ = 2–4, specifically for the changed as well as the polar bindings sites [15]. Furthermore, the results of such interactions can be based on the continuum solvation appointed creating the absolute affinities invalid or the method to be empirical. Lastly, the dielectric constant and if to improve the entropy term can also have parameters that can be modified or varied for enhancing the quality of the results.

### 2.5 Prediction of drug-like properties, bioactivity score and toxicity

The drug-like properties of selected phytocompounds were determined using the SwissADME web server. Parameters like molecular weight (MW), topological polar surface area (TPSA), number of hydrogen bond acceptors (nOHNH), number of hydrogen bond donors (nON), water partition coefficient (WLOGP), and a number of rotatable bonds (nrotb) of selected phytoconstituents were computed. The constituents were filtered based on Lipinski rule of 5 [16], Egan rule [17] and Veber’s rule [18]. Molinspiration tool [19] was used to predict the bioactivity score of filtered phytoconstituents comprising of GPCR ligands, ion channel modulators, kinase inhibitors, nuclear receptor ligands, protease inhibitors and enzyme inhibitors. Additionally, ProTox II webserver [20] was used for the analysis of the toxicity properties of selected phytoconstituents.

### 2.6 All-atoms molecular dynamic simulation

Molecular dynamic simulation of the top four complexes was performed in a simple 1-palmitoyl-2-oleoyl-sn-glycero-3-phosphocholine (POPC) lipid bilayer using Desmond. The membrane orientation of the protein was derived from the state-of-the-art tool employed in Positioning of proteins in membranes (PPM) 3.0 Web Server. Protein Preparation Wizard (Schrodinger) was used for the preparation of the receptor-ligand complexes. Acetyl and methylamide were used to cap the 5-LOX receptor, while the titratable amino acid residues were left in their dominant condition at pH 7.0. Using CHARMM-GUI Membrane Builder, each complex was embedded in a bilayer of 373POPC lipids and solvated with 0.15MNaClin explicit TIP3P waters [21]. The CHARMM36m forcefield [22] was adopted for protein, lipids and salt ions (NaCl 0.15M), while the ligand topology was adopted using CHARMM CGenFF small-molecule force field [23]. The complex system was initially relaxed using steepest descent energy minimization, then slowly heated to 310 K with restrictions. The system was simulated using the Berendsen NVT ensemble while keeping the temperature at 310 K to constrain heavy atoms on the solute. Under isothermal isobaric ensemble conditions, the MDS was done at a temperature of 300 K, a pressure of 1 atm, and a thermostat relaxation period of 200 ps (NPT). Production MD was performed for 100 ns maintaining the pressure and temperature scale at 300 K and 1 atm respectively, using Nose-Hoover thermostat and the semi-isotropic Parrinello-Rahman barostat, respectively. The snapshots from MD simulation was recorded step by step after every 50 ps. The harvested MD trajectories, which included 2000 snapshots, were then used for post-MD analysis, which included dynamics stability, flexibility, and inter-molecular contact analysis.

## 3. Results and discussion

### Structural variation and binding site determination

The solved crystal structure of the 5-LOX protein contains several mutations as well as missing residues at the N-terminal end. Therefore, we used an alphafold derived full-length model of the 5-LOXprotein structure. The structural variation of the full-length 5-LOX enzyme obtained from alphafold (green) and crystal structure 3o8y (cyan) was verified by superimposition of both the structures as shown in **S1 Fig**. It is evident from S1 Fig that the machine learning derived 5-LOX structure fits well with the experimental structure with an overall RMSD of 0.205 Å, thus signifying the overall retention of the topology in the structure derived through alphafold. In order to cross-check the suitability of side chain in the active site, we superimposed our model with the ligand-bound conformation (6N2W; 2.71 Å) of 5-Lipoxygenase, where, we observed the residues surrounding the inhibitor nordihydroguaiaretic acid (NDGA within 5 Å) perfectly superpose well with each other thereby indicating the accuracy of our model (**S2 Fig**). The binding energy of natural ligand nordihydroguaiaretic acid (NDGA) after redocking was found to be-5.25 Kcal/mol. The superimposition of the co-crystallized pose and the docking pose of the ligand nordihydroguaiaretic acid is given in S3 Fig.

A binding site represents a unique site/location on enzyme/protein that participates in the binding of certain small molecules and performs a chemical reaction. The plausible binding siteof 5-LOX with the ligands was predicted using the Prank Web server. The top three pocket score were considered as the active ligand binding sites of the protein and are presented in **S1 Table**. The first binding site had the highest pocket score, with a score value of 18.43. A total of 28 amino acids built up this pocket. The second pocket was made up of 20 amino acids and had a score value of 14.1. The third pocket had a lower binding affinity of 13.88 and was made up of 23 amino acids.

### Molecular docking studies and MM-GBSA analysis

Deciphering the mechanisms by which lipoxygenase activity occurs at the atomic level will be a benefit for designing of 5-LOX inhibitors. In this context, theoretical approach, such as molecular docking and MM/GBSA calculations were employed for investigating the molecular interactions between the ligands and enzymes. Molecular docking methods are quick and efficient tools to identify the binding of ligand molecules with the protein [15, 24]. To achieve this, the 5-LOX inhibitory potential of 23 phytocompounds was assessed by performing docking of compounds at the active site of 5-LOX. The glide ligand score shows the efficiency of ligand molecules in binding to protein 5-LOX. Glide docking score estimates the binding affinity that is directly linked to the gibbs energy of binding by taking into consideration of the entropy and enthalpy scoring functions [25]. A high negative glide score corresponds to a strong binding between the protein and ligand. At first, standard precision (SP) docking was applied to the selected 23 phytocompounds. The SP results were further filtered out using a considerably tighter and stricter docking method, extra precision (XP) docking. The XP score of 23 phytocompounds ranged from -1.73 to -5.90 kcal/mol.

**Fig 1:**
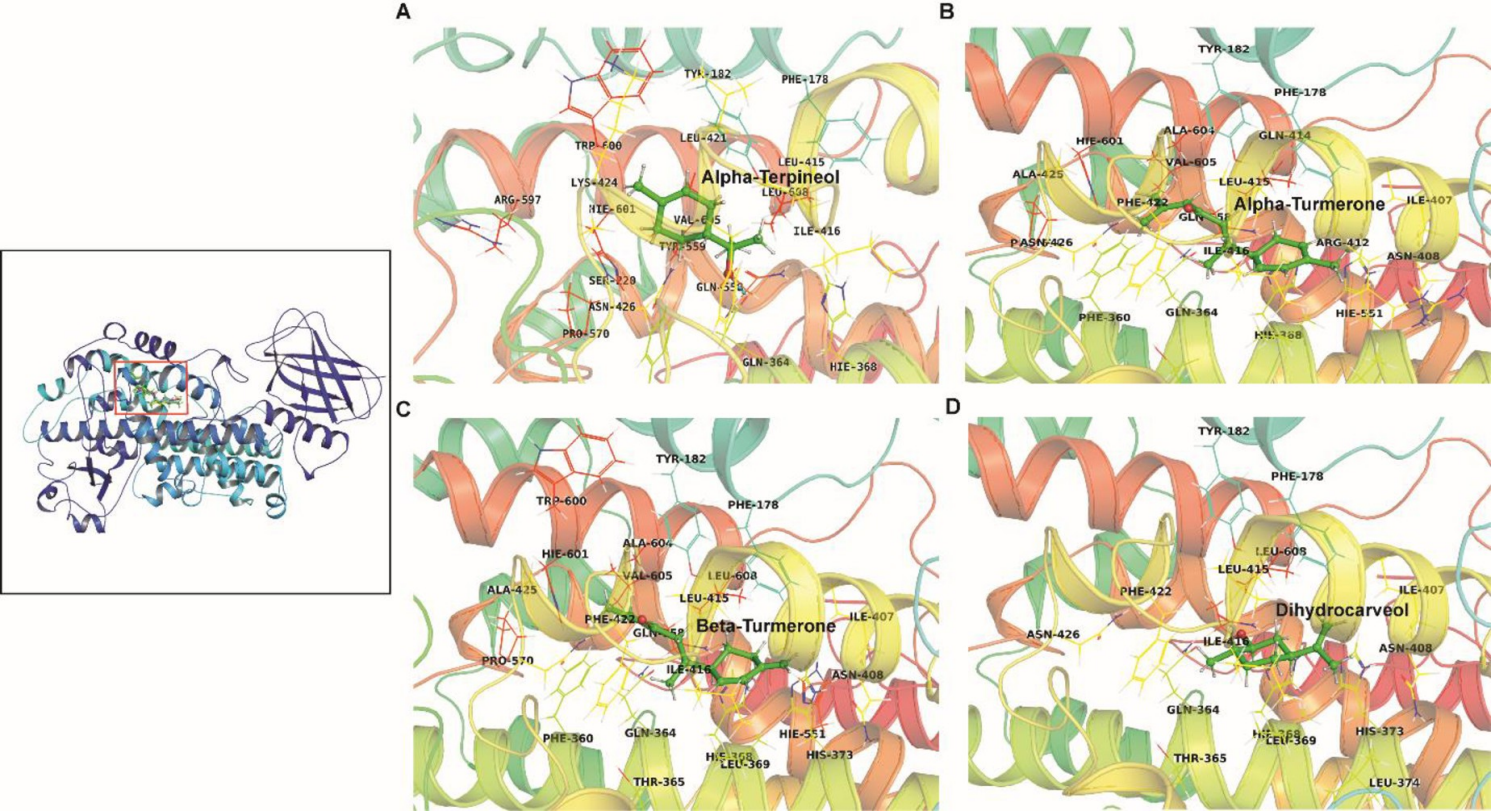
Intermolecular contact analysis of the top four-ligands (A: B: C: D:) with 5-LOX protein obtained from docking using Glide displaying various non-bonded contacts. The image was rendered using PyMOL. The hydrogen bonds are shown in dotted lines. The left panel displays the structurally superimposed view of the top ranked 5-LOX-ligand complexes obtained from docking.

The glide docking score of phytocompounds ranged from -1.73 to -5.90 kcal/mol (**Table 1**). The compounds *α*-turmerone, *β*-turmerone, *α*-terpineol and dihydrocarveol had high negative glide docking scores of -5.90, -5.70, -5.65 and -5.38 kcal/mol, respectively. Based on the binding affinity, we selected top four ligands i.e. *α*-turmerone, *β*-turmerone, *α*-terpineol and dihydrocarveol for intermolecular contact analysis as illustrated in **Fig 1** and **Table 2**. As shown in **Table 2**, Asn426, and Gln364 of 5-LOX formed two strong hydrogen bonds with the O1 and H15 atom of *α*-terpineol. Similarly, Ala425, Ala604, Lys424, and Trp600 formed alkyl hydrophobic contacts with the ligand. *α*-turmerone made dense hydrophobic contacts (Phe360, His368, Leu369, His373, Ala411, Leu415, Ile416, Phe422, Ala604, and Val605) dominated by alkyl and pi-alkyl contacts. Likewise, *α*-turmerone, *β*-turmerone also formed a complex network of hydrophobic bonds dominated by alkyl and pi-alkyl contacts where the binding site residues, *i*.*e*., Ala411, Leu369, Leu415, Ile416, Ala604, Ile407, Val605, His373, Phe178, Phe360 and Phe422 shared the same binding pocket of 5-LOX. In case of dihydrocarveol, Gln364 and His368 formed the hydrogen bond, while, Ala411, Leu369, Leu415, Ile416, Ile407, Phe178, His373 and Phe422 assisted in tight anchoring of ligand through alkyl and pi-alkyl contacts. In general, we observed that the shared binding pocket mostly forms hydrophobic contacts with the phytocompounds. Several studies reported that Ala411, His368, His373, Ile407, Ile416 and Leu369 were located around the active site, while Ala 604, Phe178, Tyr182 and Thr365 were found in the specific region of 5-LOX [26, 27].

**Table 1:** The Glide XP docking scores and MM-GBSA estimations (kcal/mol) of the compounds derived from essential oils of *Curcuma* species with 5-LOX protein (NC: Not calculated).

S. No	Ligands	PubChem CID	Glide Docking Score X(kcal/mol)	Free energy MM/GBSA (kcal/mol)
1	*α*-Farnesene	5281516	-1.73	10.75
2	*α*-Fenchol	439711	-4.57	48.52
3	*α*-Terpineol	17100	-5.65	-2.47
4	*α*-Turmerone	14632996	-5.90	-1.30
5	*α*-Zingiberene	11127403	-4.83	0.12
6	*β*-Curcumene	14014430	-5.31	4.46
7	*β*-Elemene	6918391	-3.99	60.73
8	*β*-Myrcene	31253	-2.29	-1.06
9	*β*-Ocimene	5281553	-3.16	18.34
10	*β*-Sesquiphellandrene	12315492	-4.88	6.22
11	*β*-Turmerone	9815837	-5.70	-2.43
12	Camphene	6616	-4.72	20.48
13	Curzerene	572766	-3.73	80.23
14	Curzerenone	3081930	-4.15	59.77
15	Dihydrocarveol	12072	-5.38	-2.23
16	Limonene	22311	-5.05	0.57
17	Linalool	6549	-3.87	3.15
18	Nerolidol	5284507	-2.24	0.72
19	Nerolidyl acetate	5363426	-3.05	23.49
20	Perillene	68316	-4.37	13.50
21	trans-*β*-Santalol	6450269	-3.59	32.08
22	Xanthorrhizol	93135	-5.31	5.74
23	Zingiberene	92776	-4.89	0.12

**Table 2:** Inter-molecular contact analysis portraying the different types of non-bonded contact between the top four compounds with target protein 5-LOX analyzed using BIOVIA DSV.

Ligands	Interacting pairs of atoms	Bond Length (Å)	Type of Bond	Category of Bond
*α*-Terpineol	A:ASN426:HD22—B:Ligand:O1	3.00	Hydrogen Bond	Conventional Hydrogen Bond
	B:Ligand:H15—A:GLN364:OE1	1.63	Hydrogen Bond	Conventional Hydrogen Bond
	A:ALA425—B:Ligand	4.54	Hydrophobic	Alkyl
	A:ALA425—B:Ligand:C10	3.08	Hydrophobic	Alkyl
	A:ALA604—B:Ligand	3.43	Hydrophobic	Alkyl
	B:Ligand:C10—A:LYS424	4.80	Hydrophobic	Alkyl
	A:TRP600—B:Ligand:C10	5.16	Hydrophobic	Pi-Alkyl
*α*-Turmerone	A:LEU369—B:Ligand	4.37	Hydrophobic	Alkyl
	A:ALA411—B:Ligand	4.35	Hydrophobic	Alkyl
	A:ALA411—B:Ligand:C11	3.21	Hydrophobic	Alkyl
	A:LEU415—B:Ligand	4.65	Hydrophobic	Alkyl
	A:ILE416—B:Ligand	5.34	Hydrophobic	Alkyl
	A:ALA604—B:Ligand:C15	3.54	Hydrophobic	Alkyl
	B:Ligand:C11—A:LEU369	4.61	Hydrophobic	Alkyl
	B:Ligand:C15—A:VAL605	4.10	Hydrophobic	Alkyl
	A:PHE360—B:Ligand:C14	4.91	Hydrophobic	Pi-Alkyl
	A:PHE360—B:Ligand:C15	5.04	Hydrophobic	Pi-Alkyl
	A:HIS368—B:Ligand	5.47	Hydrophobic	Pi-Alkyl
	A:HIS373—B:Ligand	4.86	Hydrophobic	Pi-Alkyl
	A:HIS373—B:Ligand:C11	3.91	Hydrophobic	Pi-Alkyl
	A:PHE422—B:Ligand:C6	4.43	Hydrophobic	Pi-Alkyl
	A:PHE422—B:Ligand:C14	5.22	Hydrophobic	Pi-Alkyl
	A:HIE601—B:Ligand:C15	4.70	Hydrophobic	Pi-Alkyl
*β*-Turmerone	A:LEU369 -: Ligand	4.98	Hydrophobic	Alkyl
	A:ALA411 -: Ligand	4.77	Hydrophobic	Alkyl
	A:ALA411 -: Ligand:C11	3.33	Hydrophobic	Alkyl
	A:LEU415 -: Ligand	4.88	Hydrophobic	Alkyl
	A:ALA604 -: Ligand:C14	3.35	Hydrophobic	Alkyl
	A:ALA604 -: Ligand:C15	3.61	Hydrophobic	Alkyl
	:Ligand:C11—A:ILE407	4.80	Hydrophobic	Alkyl
	:Ligand:C15—A:VAL605	4.02	Hydrophobic	Alkyl
	A:PHE178 -: Ligand:C11	5.13	Hydrophobic	Pi-Alkyl
	A:PHE360 -: Ligand:C15	5.34	Hydrophobic	Pi-Alkyl
	A:HIE368 -: Ligand	4.89	Hydrophobic	Pi-Alkyl
	A:HIS373 -: Ligand	4.49	Hydrophobic	Pi-Alkyl
	A:HIS373 -: Ligand:C11	3.67	Hydrophobic	Pi-Alkyl
	A:PHE422 -: Ligand:C7	4.59	Hydrophobic	Pi-Alkyl
Dihydrocarveol	:Ligand:H13—A:GLN364:OE1	1.85	Hydrogen Bond	Conventional Hydrogen Bond
	A:HIE368:HE1 -: Ligand:O1	2.72	Hydrogen Bond	Carbon Hydrogen Bond
	:Ligand:H3—A:HIE368:ND1	2.40	Hydrogen Bond	Carbon Hydrogen Bond
	A:ALA411 -: Ligand	4.78	Hydrophobic	Alkyl
	A:ALA411 -: Ligand:C9	3.48	Hydrophobic	Alkyl
	A:ALA411 -: Ligand:C10	3.87	Hydrophobic	Alkyl
	:Ligand—A:LEU369	3.73	Hydrophobic	Alkyl
	:Ligand—A:LEU415	4.72	Hydrophobic	Alkyl
	:Ligand—A:ILE416	4.79	Hydrophobic	Alkyl
	:Ligand:C9—A:LEU369	4.46	Hydrophobic	Alkyl
	:Ligand:C10—A:ILE407	4.51	Hydrophobic	Alkyl
	A:PHE178 -: Ligand:C10	4.65	Hydrophobic	Pi-Alkyl
	A:HIS373 -: Ligand:C9	3.74	Hydrophobic	Pi-Alkyl
	A:HIS373 -: Ligand:C10	3.66	Hydrophobic	Pi-Alkyl
	A:PHE422 -: Ligand:C7	4.46	Hydrophobic	Pi-Alkyl

The phytocompounds like α-terpineol and dihydrocarveol fitted well in the active pocket of 5-LOX, forming hydrogen bonds with His368 and Gln364. The α-amino group of His368 was associated with ferrous ion, making hydrogen interaction with the hydroxyl groups of *α*-terpineol and dihydrocarveol. The main influence appears to be on the ligand orientation at the active site of 5-LOX by amino acid His368, an iron-coordinating residue [3]. The compounds such as *α*-turmerone, *β*-turmerone and dihydrocarveol have hydrophobic interactions with the amino acid Phe178 that acts as cork for the active site of 5-LOX. Ligands and coordinating residues normally form a cage like structure with the iron molecule, limiting the access of another molecule to the catalytic site. The probable inhibitory action of these terpenoids could be due to their isoprene units. The chain length increases the cyclization rate based on their oxygenated functional groups, allowing for a wider range of chemical reactions to take place [28].

MM-GBSA calculation was further carried out with the docked SP and XP poses to evaluate the sensitivity to protein relaxation in the pockets (**Table 1**). MM-GBSA calculation would help to determine the predictive binding energies of the ligand. In case of MM-GSBA the binding free energy is referred to as the sum of all the intermolecular bindings that is present mainly between the ligand as well as the target. The method involves six energy terms that can be tested individually and improved, the electrostatic term is based on the charges of the receptor as well as the ligand. In these regard in order to improve the polarizable potentials, QM calculations as well as multipole expansions attempts can be taken with improvement in the ΔG bind and depends on the dielectric constant that is needed for the protein. It is to be noted that the computed binding free energy of the complexes through MM-GBSA approach doesn’t represent the true free energy as it ignores entropic contribution [29]. It is generally believed that the lower the binding value, the more stable the complex formed by the protein and the ligand. The predictive binding free energy ranges from -2.47 to 80.23 kcal/mol. *α*-terpineol exhibited the highest negative binding energy of -2.47 kcal/mol followed by *β*-turmerone (-2.43 kcal/mol). In some complexes, we also observed positive free binding energy which signifies that the compounds are not favourable for binding to the receptor 5-LOX.

**Fig 2:**
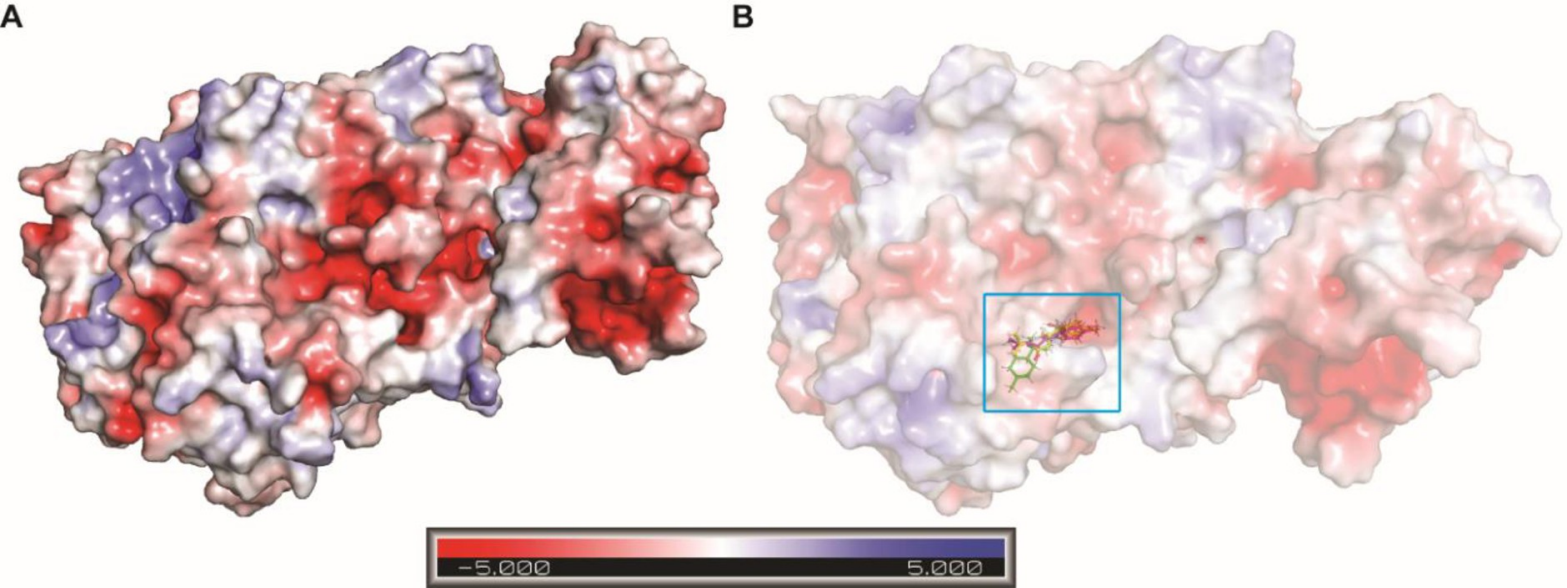
The electrostatic surface potential map of the 5-LOX protein (A) and the protein-ligand complexes (B) generated using APBS-plugin in PyMOL. Different colors like blue, red, and white epitomize positively charged, negatively charged, and neutral surface patches respectively of the protein and complexes.

In order to understand the electrostatic surface potential of the ligand binding pocket of 5-LOX, we generated the surface potential map of protein and complexes using PyMOL (**Fig 2**). Close inspection of the charge distribution within the binding pocket revealed that the cavity is surrounded by negatively charged patches (red) and neutral surface patches (white). A value of -5 kcal/mol was set as binding energy cutoff value for narrowing down the number of constituents for further *in silico* studies. Out of 23 phytocompounds, 8 compounds had a glide docking score greater than -5 kcal/mol and were considered for implementation of the second criteria of elimination by determination of ADME (absorption, distribution, metabolism and excretion) properties.

### *In silico* evaluation of ADME property, bioactivity score and toxicity parameter of selected ligands

The ADME analysis helped us to determine the physicochemical properties and drug likeness of the compound. Drug likeness would enable us to eliminate those phytocompounds which did not have a significant drug like property. The collective laws of Lipinski’s [16], Egan’s [17] and Veber’s [18] that determine the properties of a drug were followed. The rules are as follows: molecular weight (MW) <500, topological surface area (TPSA) <140, number of H-bond acceptors (nOHNH) < = 5, number of H-bond donors (nON) < = 5, water partition coefficient (WLOGP) < = 5.88, number of rotatable bonds (nrotb) < = 10. Based on these findings, a total of 7 compounds (**S2 Table**) out of 8 passed the Lipinski’s, Egan’s and Veber’s rules, thereby indicating that they possess good druglike, lead like and medicinal chemistry properties. These compounds were selected further for the calculation of bioactivity score. Additionally, the molinspiration chemoinformatics web server was used to calculate the bioactivity score in order to explore the 5-LOX enzyme inhibition of selected compounds. The bioactivity score was calculated as follows: active, moderately active, or inactive when bioactivity score is greater than 0, between − 0.5 and 0, or less than − 0.5, respectively. A value higher than zero indicates higher bioactivity of molecules [30]. Out of 7 selected phytocompounds, 5 compounds had positive bioactivity score towards the class of enzyme inhibitors (**S3 Table**). Therefore, these compounds could be considered as active and identified as potential inhibitors for targeting the 5-LOX enzyme.

We employed the web-server ProTox-II to predict the toxicity parameters of the selected phytocompounds (**S4 Table**). All the selected compounds were predicted not to be hepatotoxic, carcinogenic, immunotoxic and mutagenic. Then, to ensure the safety of the selected compounds, we also calculated the LD_50_ prediction in the **S4 Table**. All the selected phytocompounds had an LD_50_ greater than 2000 mg/kg, indicating that they are safe for biological administration and can be used as potential anti-inflammatory drugs.

### Analysis of molecular dynamic trajectories of the top scored docked complex

**Fig 3:**
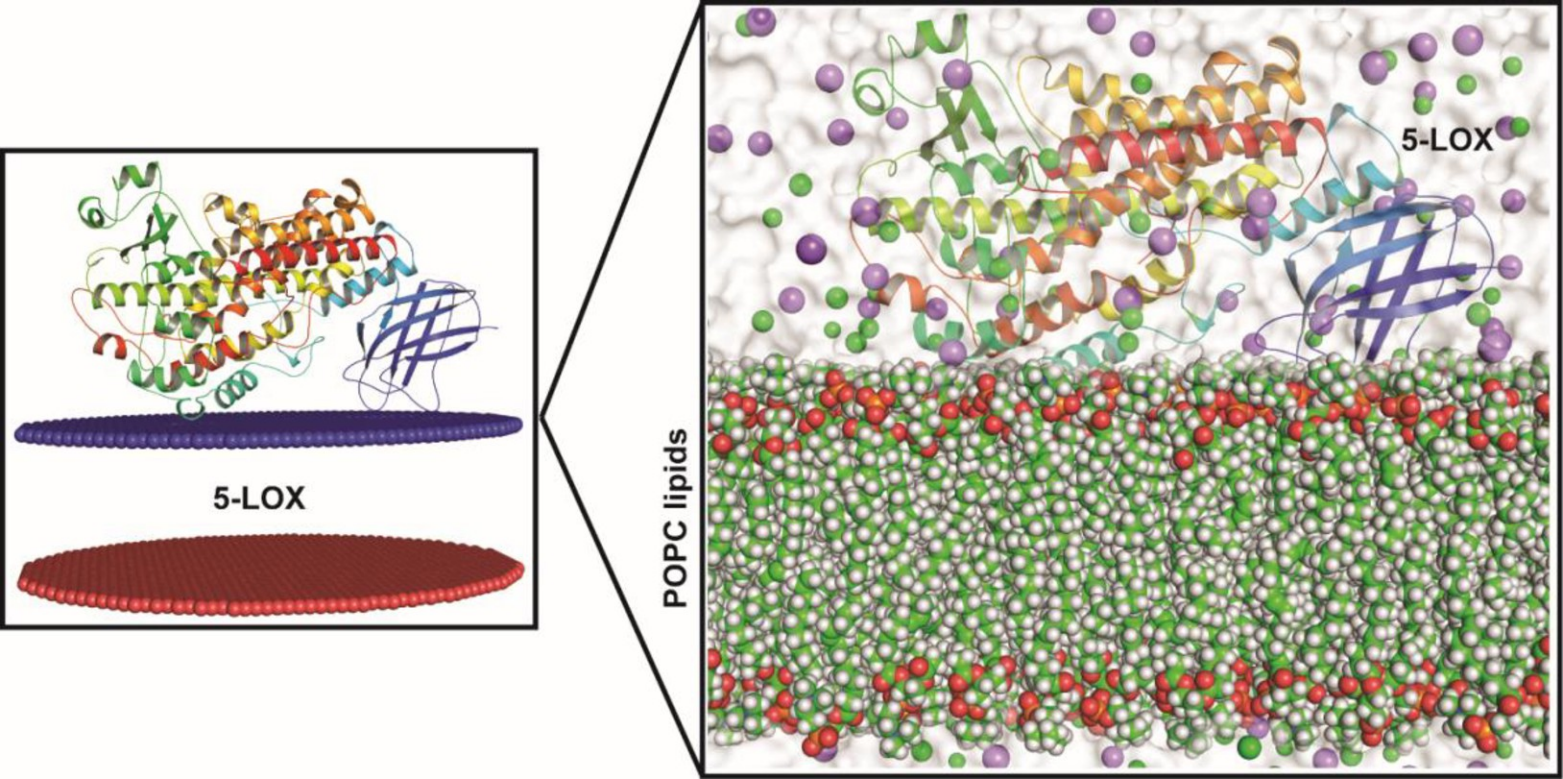
The membrane aligned view of the complete 5-LOX protein and membrane protein (shown in cartoon representation) on the surface of POPC lipid bilayer (waters shown in surface format (white), lipids in sphere format, and lipid head groups in red sphere).

**Fig 4:**
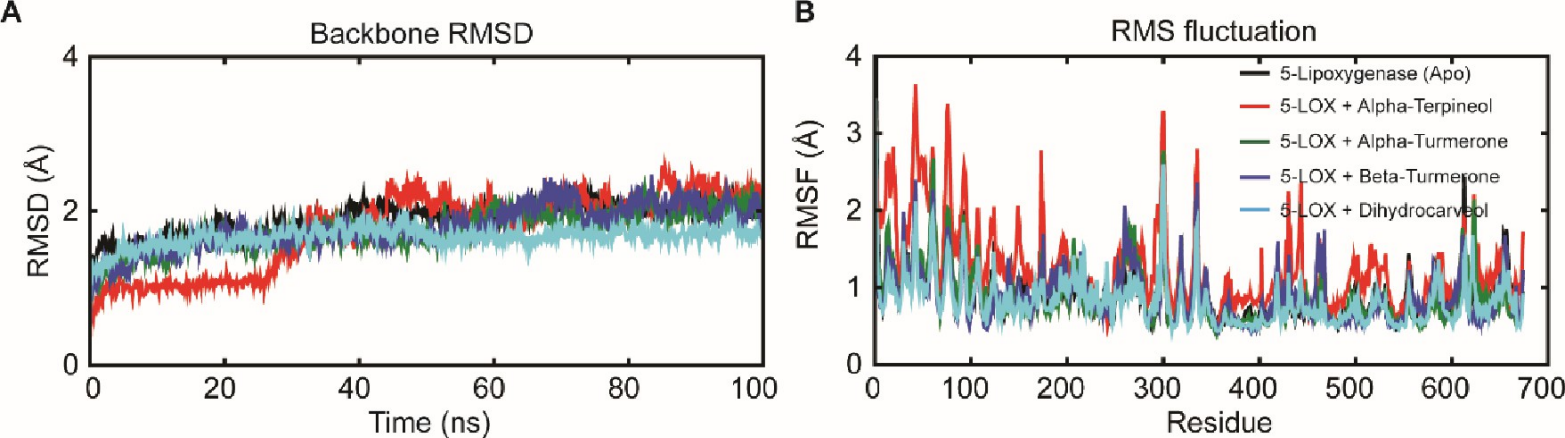
Intrinsic dynamics stability and flexibilities of the 5-LOX complex systems during all-atoms MD simulation of 100 ns. **(A)** Backbone RMSD of the five systems (including apo form and holo form of 5-LOX) membrane-water system during 500-ns MD simulations. (**B**) The Cα-root mean squared fluctuation (RMSF) profile of each amino acid of 5-LOX apo and holo systems during simulation in the lipid bilayer system.

**Fig 5:**
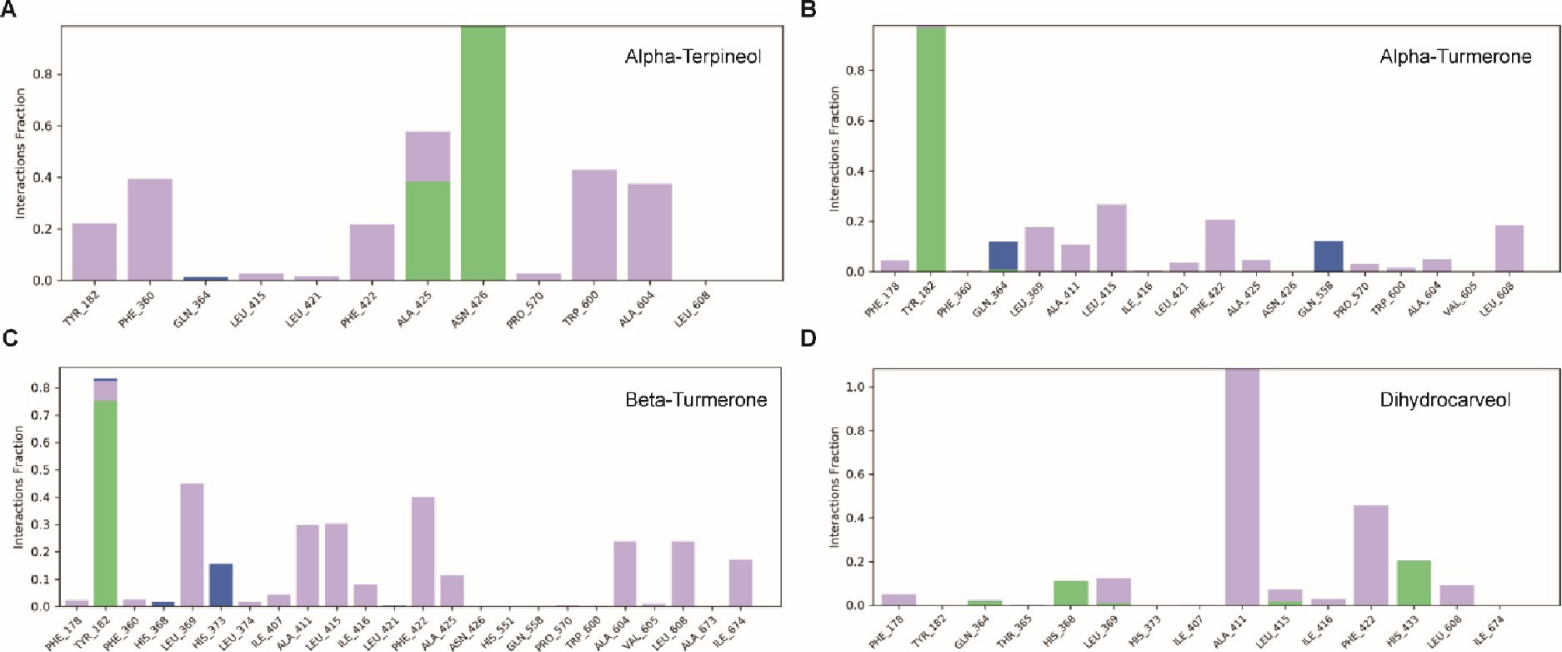
Interaction fraction of amino acids from 5-LOX complex systems involved in ligand recognition during 100 ns MD (A: B: C: D:).

Although molecular docking is a quick and efficient approach in identifying the binding pose of ligand with the active site of protein, it does not account for the conformational changes that take place during protein ligand interaction [31]. In view of this, molecular dynamics (MD) simulation is carried out to provide a more accurate estimate of conformational changes [32]. In order to understand the dynamic behavior of the 5-LOX (apo) and 5-LOX-complex (*α*-terpineol, *α*-turmerone, *β*-turmerone and dihydrocarveol), we performed all-atoms molecular dynamic (MD) simulations. The membrane aligned view of the complete 5-LOX protein and membrane protein on the surface of POPC lipid bilayer is shown in **Fig 3**. As shorter simulation run time (<50 ns) could be misleading and it would be difficult to differentiate between active and inactive ligands, simulation for 100 ns was carried out in Desmond for the five systems [(5-LOX (apo) and 5-LOX-complex (*α*-terpineol, *α*-turmerone, *β*-turmerone and dihydrocarveol)].This will help us to better understand the binding stability of the ligands inside the 5-LOX active site. The dynamic stability of each system was investigated by computing the backbone root mean square deviation (RMSD) of each protein as compared to the initial conformation. As shown in **Fig 4**, all the systems reached equilibrium after commencing simulation for 70 ns, with respect to reference frame at time 0 ns. The backbone protein displayed a stable trend in RMSD till 100 ns with an average RMSD below ~2.5 Å. Similarly, we observed a constant trend in ligand RMSD till 100 ns with an average RMSD below ~2.3Å (**S4 Fig**). This signifies that the ligand remains intact with the binding pocket as compared to docked conformation with minor changes in its orientation. The fluctuations were under the permissible range of 1–3 Å, hence can be considered as non-significant. We plotted the root mean square fluctuation (RMSF) profile of the C-α atom of all the systems to infer any local changes in 5-LOX holo systems upon phytocompounds binding compared to apo systems. The N-terminal end of all the systems displayed a high degree of fluctuation, where, the amino acid residues involved in interaction with the compounds showed remarkable changes (with higher RMSF) as compared to their apo state, thereby indicating their participation in ligand recognition. The RMSF of each amino acid of 5-LOX apo and holo systems ranged from 0.7 to 3.73 Å. 5-LOX-*α*-terpineol displayed higher fluctuations as compared to the other complex and apo systems. Comparative analysis of the initial structure with the MD simulated structure of 5-LOX revealed that the structural integrity of the model remained intact during simulation, which signifies that our system captures the true dynamics of the protein and complexes in lipid bilayer (**S5 Fig**). In addition, the interactions between phytocompounds and the amino acid of 5-LOX were monitored for 100 ns to generate the interaction potential map (**Fig 5**). The hydrogen-bond, hydrophobic contact, and ionic interaction are shown in interaction fraction in the Y-axis and the residues that aid in interaction are displayed in X-axis (where the value >1.0 indicates multiple contacts with small molecules). Though we observed minor changes in the interaction of phytocompounds with 5-LOX during simulation, the important bonds that were observed in the initial ligand docked compounds did not alter during the course of simulation.

**Fig 6:**
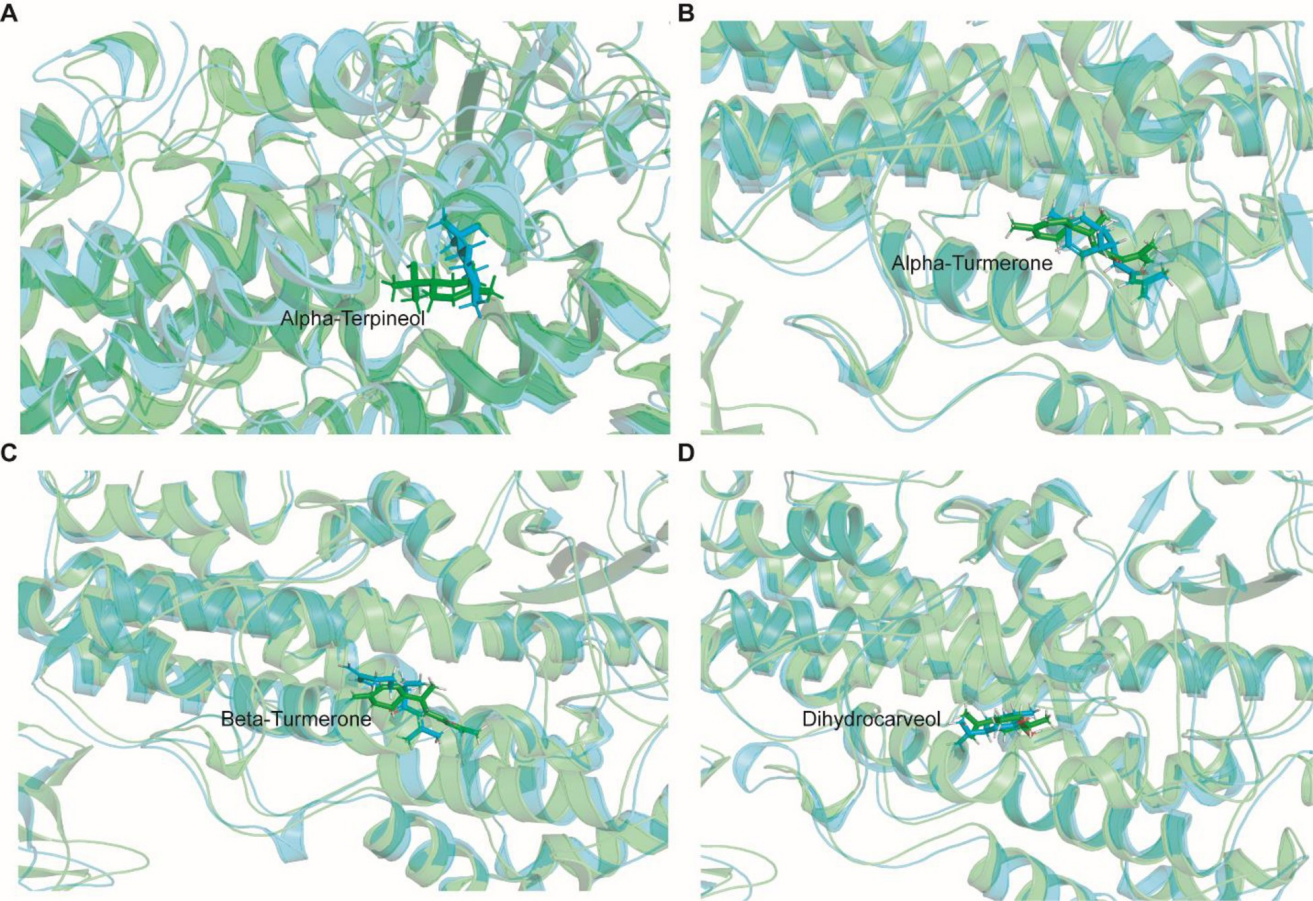
Structural superimposed view of the pre (green) and post-MD (cyan) snapshots displaying the orientation of ligands within the binding pocket of 5-LOX protein.

**Fig 7:**
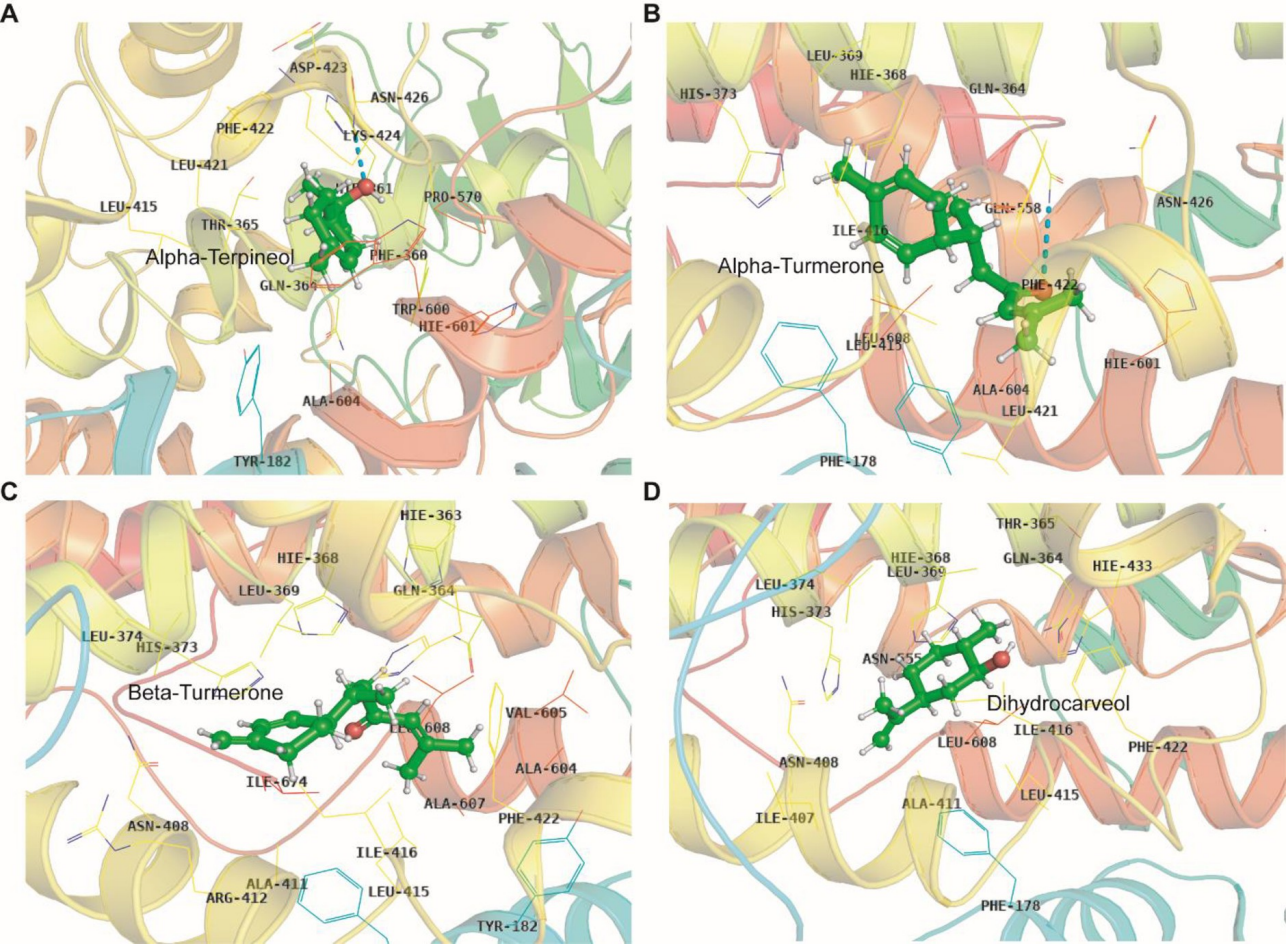
Interaction of ligands with 5-LOX protein obtained from the MD trajectories upon clustering. The protein is shown in cartoon format and the ligands are shown in ball-stick format. The interacting amino acids are labelled in black.

Besides the number of hydrogen bonds, the changes in the ligand orientation that helps in minimizing hydrophobic interactions may also play a potential role in ligand recognition (**Figs 6 and 7**). Cross-comparison of the docked conformation with that of representative structures from top ranked clusters (obtained from MD trajectories) clearly demonstrated that the ligand prefers to remain in the same binding pocket with minor changes in their orientation, which confirms the accuracy of the docking protocol employed in this study. The ligand binding affinities can be represented by MM-GBSA.

Further, in order to understand the changes in the secondary structure elements in the apo and holo states of the enzyme, we used the timeline module of VMD to plot the evolution of secondary structure elements (**S6** and **S7 Figs**). In all systems, the most important fold of the enzyme remained stable with a minor change in the loop region. A significant change in the ligand binding region of the 5-LOX complex systems was observed as compared to the apo system. The interaction of MD trajectories demonstrated the stability of interaction and the conformational changes at different points of the simulation.

## 4. Conclusion

The present study demonstrated the inhibitory potential of bioactive constituents of essential oils of *Curcuma* species against 5-LOX through computational approaches. The compounds such as *α*-turmerone, *β*-turmerone, *α*-terpineol and dihydrocarveol were the best lead compounds to target the pro-inflammatory enzyme5-LOX.Additionally, all atom MD simulations conducted on these 4 phytocompounds confirmed that the selected compounds bind to the primary binding site of 5-LOX and induces a small conformational change in the binding site, which enables the ligands to reorient within the binding interface. To the best of our knowledge, this is the first ever computational study which has uncovered four promising anti-inflammatory bioactive compounds from essential oils of *Curcuma* species with higher binding affinity against 5-LOX. However, further *in vitro* and *in vivo* validation is required for development of novel anti-inflammatory drugs for treatment of asthma and other inflammatory diseases in humans.

## Supporting information

S1 Table PrankWeb result summary of 5-LOXwith the prospective pockets and the expected amino acids. (DOCX)

S2 Table Drug-like qualities of the 7 phytocompounds that passed the Lipinski rule of 5, Veber’s rule, and Egan rule. (DOCX)

S3 Table Bioactivity score of the selected 5 phytocompounds derived from Molinspiration. (DOCX)

S4 Table Prediction of toxicity evaluated with program Protox. (DOCX)

S1 Fig Structural-superimposed view of the full-length 5-LOX enzyme derived from alphafold (green) and crystal structure 3o8y (cyan). (DOCX)

S2 Fig Superimposed 3-D structure of alphafold model and ligand-bound 5-Lipoxygenase protein (6N2W) as viewed by Pymol. Green color indicates alphafold model protein (3O8Y) whereas red color indicateligand-bound 5-Lipoxygenaseprotein (6N2W).
(DOCX)

S3 Fig Superimposition of the co-crystallized pose and the docking pose of the ligand nor-dihydroguaiaretic acid. (DOCX)

S4 Fig The trend in RMSD of the ligands in 5-LOX complexes during all-atoms MD simulation in lipid bilayers for 100 ns. (DOCX)

S5 Fig Structural superimposed view of the 5-LOX protein (before MD: Green) with MD simulated protein after 100 ns seconds (cyan). (DOCX)

S6 Fig The evolution of secondary structure elements of 5-LOX protein during 100 ns production MD in lipid bilayers. The plot was generated using timeline module of VMD.
(DOCX)

S7 Fig The development of secondary structural components of 5-LOX proteins *α*-Terpineol, *α*-turmerone, *β*-turmerone, and dihydrocarveol over 100 ns production MD in lipid bilayers. The plot was created with VMD’s timeline module.
(DOCX)
