# Supplementary material for: ‘Mechanistic insights into 5-lipoxygenase inhibition by active principles derived from essential oils of Curcuma species: Molecular docking, ADMET analysis and molecular dynamic simulation study
Source: PLoS One. 2022 Jul 22;17(7):e0271956. doi: 10.1371/journal.pone.0271956 (PMC9307165; doi:10.1371/journal.pone.0271956)
Supplement: S1 Table — (DOCX) [file pone.0271956.s001.docx]

**Table S1.**PrankWeb result summary of 5-LOXwith the prospective pockets and the expected amino acids

| Pocket | Amino acids make up the pocket | Grid center | | |
| --- | --- | --- | --- | --- |
|  |  | X | Y | Z |
| 1 | Gln33, Tyr118, Arg119, Trp120, Asp184, Ile185, Gln186, Phe187, Asp188, Gly192, Val193, Tyr401, Val415, Ala416, Val418, Arg419, Phe420, Gln629, Asn631, Glu632, Leu633, Met637, Pro639, Glu640, Asn687, Ser688, Ala690, Tyr99 | 9.6695 | 13.6686 | -11.3736 |
| 2 | Pro260, Val261, Leu262, Arg264, Asp303, Glu305, Leu306, Ile383, Arg388, Ala457, Asp460, Ser465, Leu466, Phe468, Ala471, Ile472, Arg475, Phe562, Thr563, Gln567 | 11.2625 | 31.7008 | 7.7301 |
| 3 | Pro116, Arg119, Trp120, Thr122, Asp124, Val125, Glu126, Val128, Leu129, Arg130, His148, Glu152, Thr155, Arg156, Gln159, Tyr160, Pro182, Arg183, Asp184, Tyr401, Ala406, Val407 | 14.1964 | 20.1479 | -24.2572 |
