# Supplementary material for: ‘Mechanistic insights into 5-lipoxygenase inhibition by active principles derived from essential oils of Curcuma species: Molecular docking, ADMET analysis and molecular dynamic simulation study
Source: PLoS One. 2022 Jul 22;17(7):e0271956. doi: 10.1371/journal.pone.0271956 (PMC9307165; doi:10.1371/journal.pone.0271956)
Supplement: S2 Table — (DOCX) [file pone.0271956.s002.docx]

**Table S2.** Drug-like qualities of the 7 phytocompounds that passed the Lipinski rule of 5, Veber's rule, and Egan rule

| S No. | Phytocompounds | MW (<500) | TPSA (<=140) | nOHNH  (<=5) | nON (<=5) | WLOGP (<=5.88) | nrotb (<=10) |
| --- | --- | --- | --- | --- | --- | --- | --- |
| 1 | *α*-Terpineol | 154.25 | 20.23 | 1 | 1 | 2.5 | 1 |
| 2 | *α*-Turmerone | 218.33 | 17.07 | 1 | 0 | 4.07 | 4 |
| 3 | *β*-Curcumene | 204.35 | 0 | 0 | 0 | 5.04 | 4 |
| 4 | *β*-Turmerone | 218.33 | 17.07 | 1 | 0 | 4.07 | 4 |
| 5 | Dihydrocarveol | 154.25 | 20.23 | 1 | 1 | 2.36 | 1 |
| 6 | Limonene | 136.23 | 0 | 0 | 0 | 3.31 | 1 |
| 7 | Xanthorrhizol | 218.33 | 20.23 | 1 | 1 | 4.55 | 4 |
