# Supplementary material for: ‘Mechanistic insights into 5-lipoxygenase inhibition by active principles derived from essential oils of Curcuma species: Molecular docking, ADMET analysis and molecular dynamic simulation study
Source: PLoS One. 2022 Jul 22;17(7):e0271956. doi: 10.1371/journal.pone.0271956 (PMC9307165; doi:10.1371/journal.pone.0271956)
Supplement: S3 Table — (DOCX) [file pone.0271956.s003.docx]

**Table S3.**Bioactivity score of the selected 5 phytocompounds derived from Molinspiration

| S No. | Phytocompounds | GPCR ligand | Ion channel modulator | Kinase inhibitor | Nuclear receptor ligand | Protease inhibitor | Enzyme inhibitor |
| --- | --- | --- | --- | --- | --- | --- | --- |
| 1 | *α*--Terpineol | -0.51 | 0.15 | -1.45 | -0.02 | -0.78 | 0.14 |
| 2 | *α*-Turmerone | -0.48 | -0.2 | -1.31 | 0.51 | -0.55 | 0.39 |
| 3 | *β*-Curcumene | -0.2 | -0.03 | -0.77 | 0.21 | -0.61 | 0.33 |
| 4 | *β*-Turmerone | -0.47 | -0.23 | -1.42 | 0.58 | -0.5 | 0.35 |
| 5 | Dihydrocarveol | -0.62 | 0.25 | -1.54 | -0.26 | -0.56 | 0.06 |
