# Supplementary material for: ‘Mechanistic insights into 5-lipoxygenase inhibition by active principles derived from essential oils of Curcuma species: Molecular docking, ADMET analysis and molecular dynamic simulation study
Source: PLoS One. 2022 Jul 22;17(7):e0271956. doi: 10.1371/journal.pone.0271956 (PMC9307165; doi:10.1371/journal.pone.0271956)
Supplement: S4 Table — (DOCX) [file pone.0271956.s004.docx]

**Table S4.**Prediction of toxicity evaluated with program Protox

| Phytocompound | Predicted LD_50_ mg/kg | Predicted Toxicity Class | Hepatotoxicity | Carcinogenicity | Immunotoxicity | Mutagenicity | Cytotoxicity |
| --- | --- | --- | --- | --- | --- | --- | --- |
| *α*-Turmerone | 10000 | 6 | Inactive 0.7 | Inactive 0.7 | Inactive 0.86 | Inactive 0.93 | Inactive 0.81 |
| *β*-Turmerone | 4600 | 5 | Inactive 0.71 | Inactive 0.8 | Inactive 0.81 | Inactive 0.94 | Inactive 0.8 |
| Dihydrocarveol | 5000 | 5 | Inactive 0.71 | Inactive 0.79 | Inactive 0.99 | Inactive 0.87 | Inactive 0.93 |
| *α*-Terpineol | 2830 | 5 | Inactive 0.72 | Inactive 0.76 | Inactive 0.99 | Inactive 0.9 | Inactive 0.64 |
| *β*-Curcumene | 3650 | 5 | Inactive 0.82 | Inactive 0.76 | Inactive 0.98 | Inactive 0.91 | Inactive 0.82 |
