# Supplementary material for: ‘Mechanistic insights into 5-lipoxygenase inhibition by active principles derived from essential oils of Curcuma species: Molecular docking, ADMET analysis and molecular dynamic simulation study
Source: PLoS One. 2022 Jul 22;17(7):e0271956. doi: 10.1371/journal.pone.0271956 (PMC9307165; doi:10.1371/journal.pone.0271956)
Supplement: S1 Fig — (DOCX) [file pone.0271956.s005.docx]

**
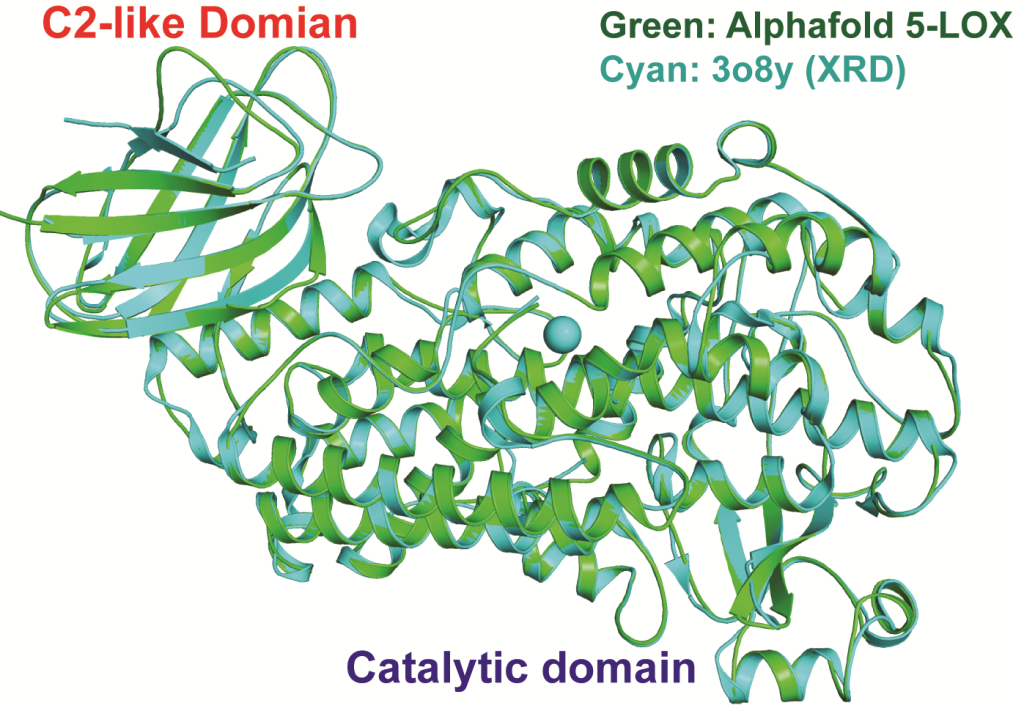
**

**Figure S1.** Structural-superimposed view of the full-length 5-LOX enzyme derived from alphafold(green) and crystal structure 3o8y (cyan)
