# Supplementary material for: ‘Mechanistic insights into 5-lipoxygenase inhibition by active principles derived from essential oils of Curcuma species: Molecular docking, ADMET analysis and molecular dynamic simulation study
Source: PLoS One. 2022 Jul 22;17(7):e0271956. doi: 10.1371/journal.pone.0271956 (PMC9307165; doi:10.1371/journal.pone.0271956)
Supplement: S2 Fig — Green color indicates alphafold model protein (3O8Y) whereas red color indicateligand-bound 5-Lipoxygenaseprotein (6N2W). (DOCX) [file pone.0271956.s006.docx]

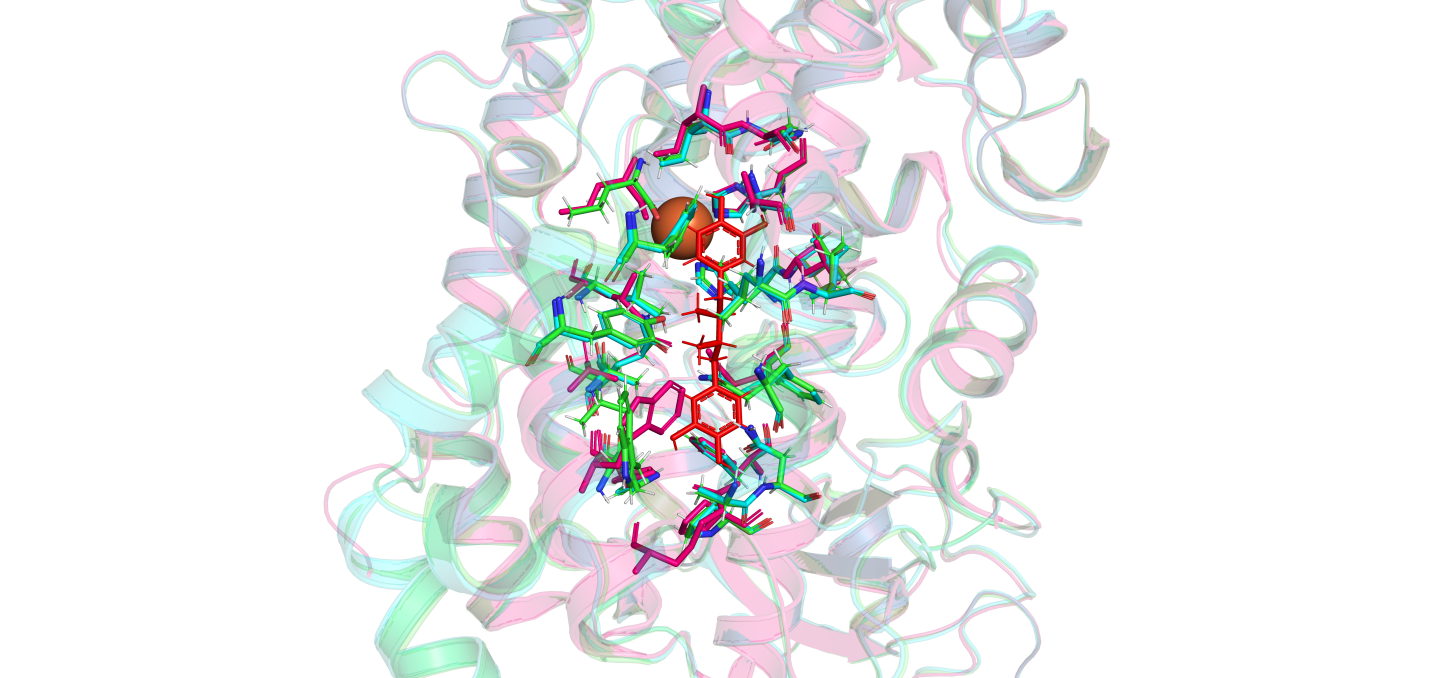


**Figure S2.** Superimposed 3-D structure of alphafold model and ligand-bound 5-Lipoxygenase protein (6N2W) as viewed by Pymol. Green color indicates alphafold model protein (3O8Y) whereas red color indicateligand-bound 5-Lipoxygenaseprotein (6N2W).
