# Supplementary material for: ‘Mechanistic insights into 5-lipoxygenase inhibition by active principles derived from essential oils of Curcuma species: Molecular docking, ADMET analysis and molecular dynamic simulation study
Source: PLoS One. 2022 Jul 22;17(7):e0271956. doi: 10.1371/journal.pone.0271956 (PMC9307165; doi:10.1371/journal.pone.0271956)
Supplement: S3 Fig — (DOCX) [file pone.0271956.s007.docx]

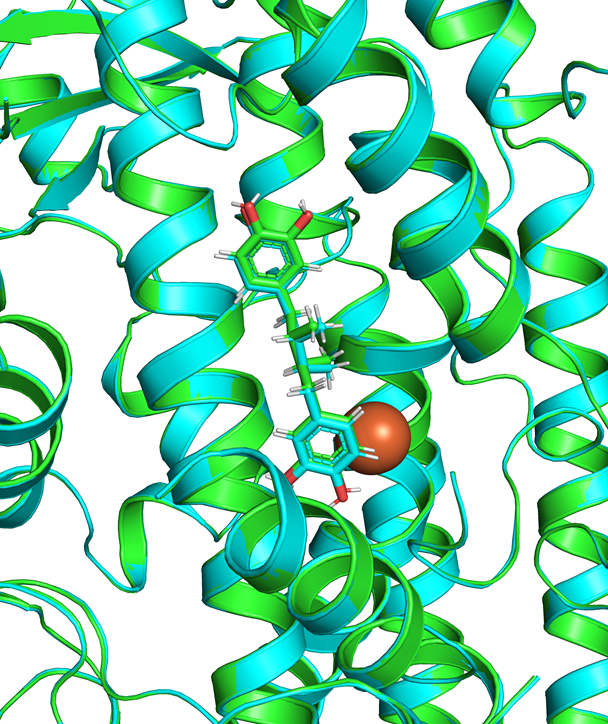


**Figure S3.** Superimposition of the co-crystallized pose and the docking pose of the ligand nor-dihydroguaiaretic acid.
