# Supplementary material for: ‘Mechanistic insights into 5-lipoxygenase inhibition by active principles derived from essential oils of Curcuma species: Molecular docking, ADMET analysis and molecular dynamic simulation study
Source: PLoS One. 2022 Jul 22;17(7):e0271956. doi: 10.1371/journal.pone.0271956 (PMC9307165; doi:10.1371/journal.pone.0271956)
Supplement: S4 Fig — (DOCX) [file pone.0271956.s008.docx]

**
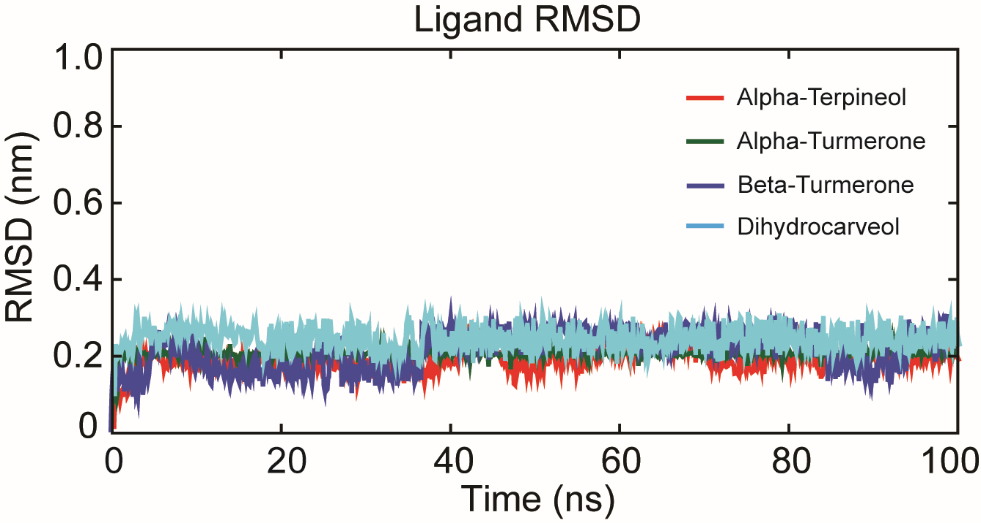
**

**Figure S4.** The trend in RMSD of the ligands in 5-LOX complexes during all-atoms MD simulation in lipid bilayers for 100 ns.
