# Supplementary material for: ‘Mechanistic insights into 5-lipoxygenase inhibition by active principles derived from essential oils of Curcuma species: Molecular docking, ADMET analysis and molecular dynamic simulation study
Source: PLoS One. 2022 Jul 22;17(7):e0271956. doi: 10.1371/journal.pone.0271956 (PMC9307165; doi:10.1371/journal.pone.0271956)
Supplement: S5 Fig — (DOCX) [file pone.0271956.s009.docx]

**
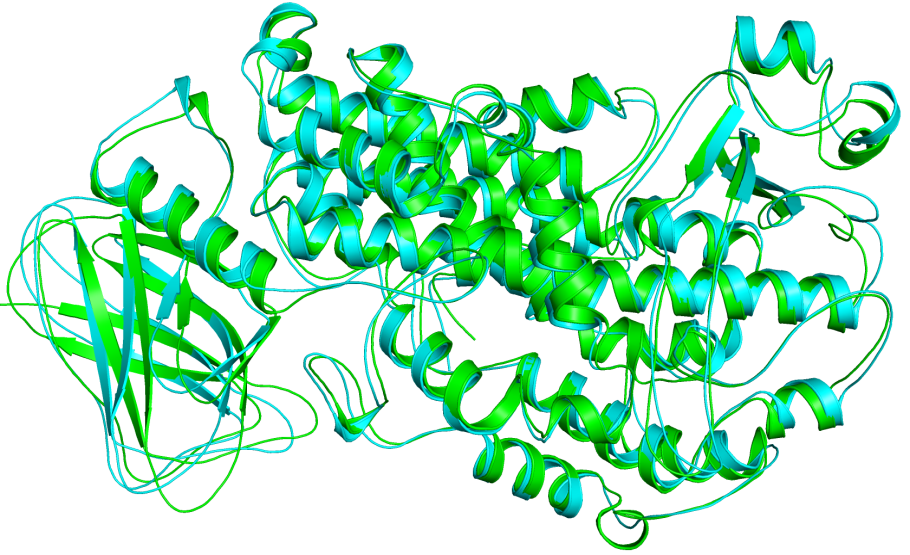
**

**Figure S5.** Structural superimposed view of the 5-LOX protein (before MD: green) with MD simulated protein after 100 ns seconds (cyan).
