# Supplementary material for: ‘Mechanistic insights into 5-lipoxygenase inhibition by active principles derived from essential oils of Curcuma species: Molecular docking, ADMET analysis and molecular dynamic simulation study
Source: PLoS One. 2022 Jul 22;17(7):e0271956. doi: 10.1371/journal.pone.0271956 (PMC9307165; doi:10.1371/journal.pone.0271956)
Supplement: S7 Fig — The plot was created with VMD’s timeline module. (DOCX) [file pone.0271956.s011.docx]

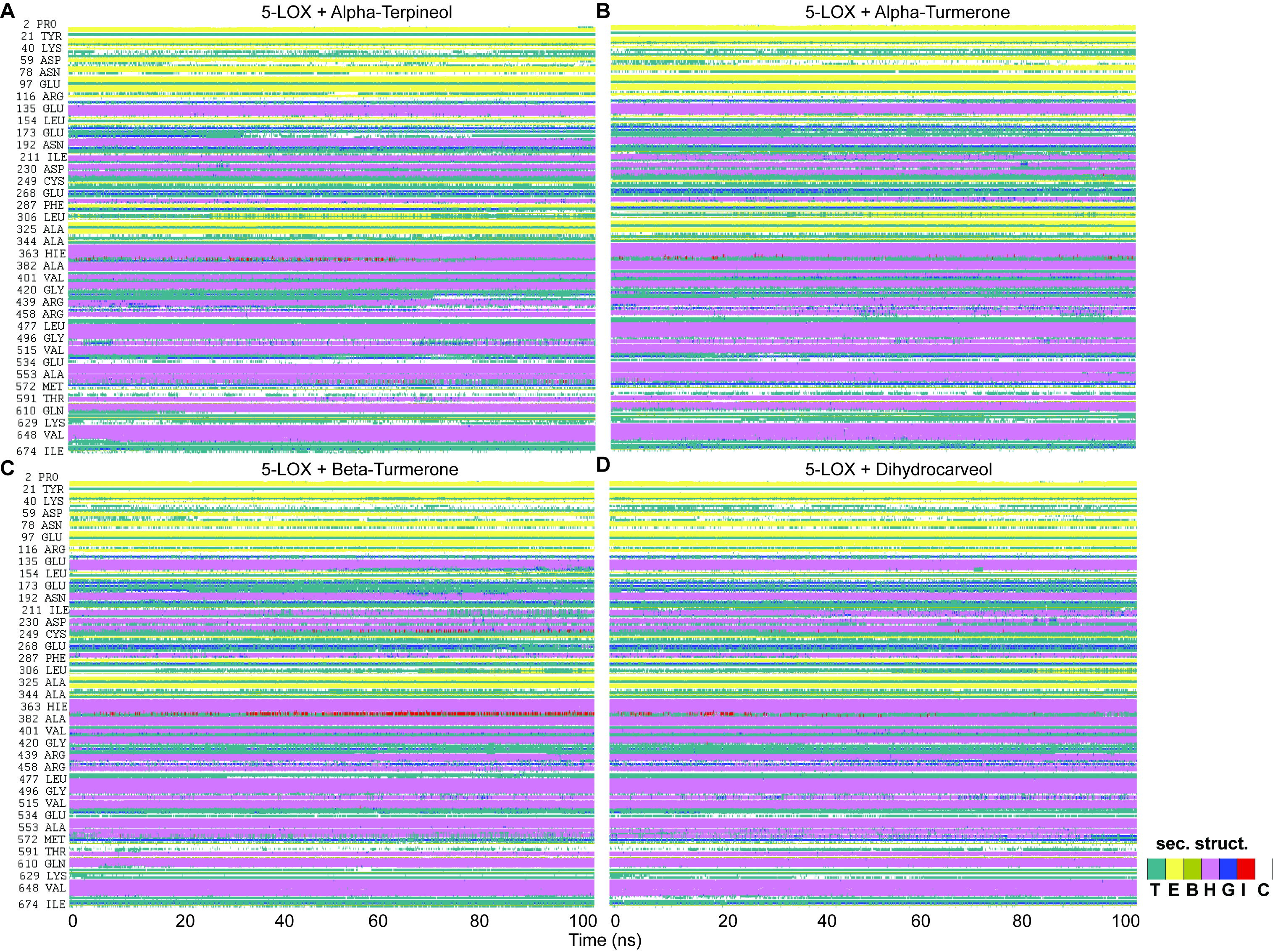


**Figure S7.** The development of secondary structural components of 5-LOX proteins *α*-Terpineol, *α*-turmerone, *β*-turmerone, and dihydrocarveol over 100 ns production MD in lipid bilayers. The plot was created with VMD's timeline module.
